# Twisted Gastrulation, a BMP Antagonist, Exacerbates Podocyte Injury

**DOI:** 10.1371/journal.pone.0089135

**Published:** 2014-02-25

**Authors:** Sachiko Yamada, Jin Nakamura, Misako Asada, Masayuki Takase, Taiji Matsusaka, Taku Iguchi, Ryo Yamada, Mari Tanaka, Atsuko Y. Higashi, Tomohiko Okuda, Nariaki Asada, Atsushi Fukatsu, Hiroshi Kawachi, Daniel Graf, Eri Muso, Toru Kita, Takeshi Kimura, Ira Pastan, Aris N. Economides, Motoko Yanagita

**Affiliations:** 1 Department of Nephrology, Graduate School of Medicine, Kyoto University, Kyoto, Kyoto, Japan; 2 Department of Internal Medicine, Tokai University School of Medicine, Isehara, Kanagawa, Japan; 3 TMK Project, Kyoto University Graduate School of Medicine, Kyoto, Kyoto, Japan; 4 Deaprtment of Cardiovascular Medicine, Graduate School of Medicine, Kyoto University, Kyoto, Kyoto, Japan; 5 Yachiyo Hospital, Anjyo, Aichi, Japan; 6 Department of Cell Biology, Institute of Nephrology, Niigata University Graduate School of Medical and Dental Sciences, Niigata, Niigata, Japan; 7 Institute of Oral Biology, Faculty of Medicine, University of Zurich, Zurich, Switzerland; 8 Department of Nephrology and Dialysis, Kitano Hospital, Tazuke Kofukai Medical Research Institute, Osaka, Osaka, Japan; 9 Kobe City Medical Center General Hospital, Kobe, Hyogo, Japan; 10 Laboratory of Molecular Biology, Center for Cancer Research, National Cancer Institute, National Institutes of Health, Bethesda, Maryland, United States of America; 11 Regeneron Pharmaceuticals, Inc., Tarrytown, New York, United States of America; Fondazione IRCCS Ospedale Maggiore Policlinico & Fondazione D'Amico per la Ricerca sulle Malattie Renali, Italy

## Abstract

Podocyte injury is the first step in the progression of glomerulosclerosis. Previous studies have demonstrated the beneficial effect of bone morphogenetic protein 7 (Bmp7) in podocyte injury and the existence of native Bmp signaling in podocytes. Local activity of Bmp7 is controlled by cell-type specific Bmp antagonists, which inhibit the binding of Bmp7 to its receptors. Here we show that the product of *Twisted gastrulation* (Twsg1), a Bmp antagonist, is the central negative regulator of Bmp function in podocytes and that *Twsg1* null mice are resistant to podocyte injury. Twsg1 was the most abundant Bmp antagonist in murine cultured podocytes. The administration of Bmp induced podocyte differentiation through Smad signaling, whereas the simultaneous administration of Twsg1 antagonized the effect. The administration of Bmp also inhibited podocyte proliferation, whereas simultaneous administration of Twsg1 antagonized the effect. Twsg1 was expressed in the glomerular parietal cells (PECs) and distal nephron of the healthy kidney, and additionally in damaged glomerular cells in a murine model of podocyte injury. *Twsg1* null mice exhibited milder hypoalbuminemia and hyperlipidemia, and milder histological changes while maintaining the expression of podocyte markers during podocyte injury model. Taken together, our results show that Twsg1 plays a critical role in the modulation of protective action of Bmp7 on podocytes, and that inhibition of Twsg1 is a promising means of development of novel treatment for podocyte injury.

## Introduction

Podocytes have recently emerged as an early injury site in many types of kidney disease. Podocyte loss correlates with severity of glomerular injury and degree of proteinuria, and leads to glomerular sclerosis[1], [2], [3], [4], [5], [6], [7]. Thus the development of therapeutic techniques attenuating podocyte injury is expected to retard the progression of kidney disease.

Bone morphogenetic protein 7 (Bmp7) is a member of the Bmp family within the TGF-β superfamily, and plays pivotal roles in the development of the kidneys and eyes[8], [9]. While Bmp7 is widely expressed during development, its expression in most tissues decreases after birth, and the kidney becomes the main site of Bmp7 production among adult tissues. In the adult kidney, Bmp7 is highly expressed in podocytes, distal tubules, and collecting ducts[10], whereas native Bmp signaling in the healthy kidney occurs mainly in podocytes and collecting ducts[11].

Recently, several groups have demonstrated that systemically administered Bmp7 retards the progression of glomerular diseases[12], [13], [14]. Some studies more specifically documented the beneficial effect of Bmp7 in the prevention of podocyte injury utilizing *Bmp7* transgenic mice[15] and cultured podocytes[16], [17], indicating the essential role of Bmp7 in the maintenance of podocyte structure and function. Little is known, however, about the potential factors regulating endogenous Bmp7 activity in podocytes.

The local activity of endogenous Bmp is controlled by certain classes of binding molecules that act as positive or negative regulators of Bmp signaling[18], [19], [20], [21], [22]. Bmp antagonists function through direct association with Bmp, inhibiting the binding of Bmp to its receptors. Previously we identified the product of *uterine sensitization–associated gene-1* (USAG-1) as a Bmp antagonist, which is by far the most abundantly expressed in the kidney, and demonstrated that USAG-1 negatively regulates the renoprotective activity of Bmp7 in many types of kidney disease[23], [24], [25], [26]. USAG-1 co-localizes with Bmp7 in the distal tubules, but is not expressed in podocytes[25]. Existence of endogenous Bmp signaling in podocytes led us to search for a Bmp antagonist expressed in podocytes.

Twisted gastrulation (Twsg1) is a Bmp modulator that synergistically interacts with chordin or chordin-like molecules to regulate Bmp activity[27]. Twsg1 can modulate Bmp activity in a positive or negative manner depending on the context[18], [27], [28], [29], [30], [31], [32]. Previously we have shown that Twsg1 is the second most abundant Bmp antagonist in the kidney next to USAG-1[24]; nevertheless, the function of Twsg1 during kidney disease progression remains to be elucidated.

Here, we demonstrate that Twsg1 plays an essential role in the progression of podocyte injury, possibly by antagonizing the renoprotective function of Bmp7.

## Methods

### Animals

Heterozygote *Twsg1-lacZ* reporter mice (*Twsg1^+/lacZ^* mice)[33] and NEP mice[34] have been described elsewhere. The background strain for the NEP25 mice was C57BL/6. *Twsg1^LacZ/LacZ^* pups were significantly fewer than expected in C57BL/6 background, possibly due to the embryonic lethality. To obtain enough number of *Twsg1^LacZ/LacZ^* mice, we utilized the mixed background between C57BL/6 and 129/Svj strains in *Twsg1^LacZ/+^* mice. To minimize the background effect, all experiments were performed with *Twsg1^+/+^:NEP25* littermates and *Twsg1^LacZ/LacZ^:NEP25* littermates. All animal studies were approved by the Animal Research Committee, Graduate School of Medicine, Kyoto University, and were strictly in accordance with the Guide for the Care and Use of Laboratory Animals of the National Institutes of Health.

### Cell Cultures

Conditionally immortalized murine podocytes were kind gifts from Professor Mundel (Mt. Sinai School of Medicine, New York) and Professor Shankland (Division of Nephrology, Department of Medicine, University of Washington). Podocytes were cultured and differentiated as previously described[35], [36]. Under permissive conditions, the podocytes from Professor Mundel were seeded at a concentration of 5×10^3^/ml and grown at 33°C in collagen I-coated culture dishes in DMEM containing 10% FCS and 10 U/ml of IFN-γ. The podocytes from Professor Shankland were seeded at a concentration of 2×10^4^/ml and grown at 33°C in collagen I-coated culture dishes in RPMI 1640 containing 10% FCS and 50 U/ml of IFN-γ. To induce differentiation, podocytes were incubated at 37°C without IFN-γ for 7–14 days. While only the results obtained using the podocytes from Professor Mundel are shown in the figures, the expression pattern of Bmp and Bmp antagonists in the two groups of podocytes was similar.

### Cell treatments

For a study of the effect of Bmps on podocyte differentiation, podocytes were incubated under non-permissive conditions. Recombinant proteins (300 ng/ml) were added to the culture medium at 24 h and on day 7, then incubated for an additional 7 days before analysis. We utilized recombinant human Bmp4, Bmp7, and Twsg1 (R&D Systems, Minneapolis, MN, USA). For a study of dorsomorphin, podocytes were incubated with 0.5–2 µM dorsomorphin (Calbiochem, Darmstadt, Germany) or dimethyl sulfoxide (DMSO, negative control) in the presence or absence of Bmp7, and were harvested 7 days later.

For a study of Bmp signaling pathways, podocytes treated with Bmp and Twsg1 were subjected to western blotting utilizing antibodies against pSmad1/5 (Cell Signaling) and GAPDH (Fitzgerald).

**Figure 1:**
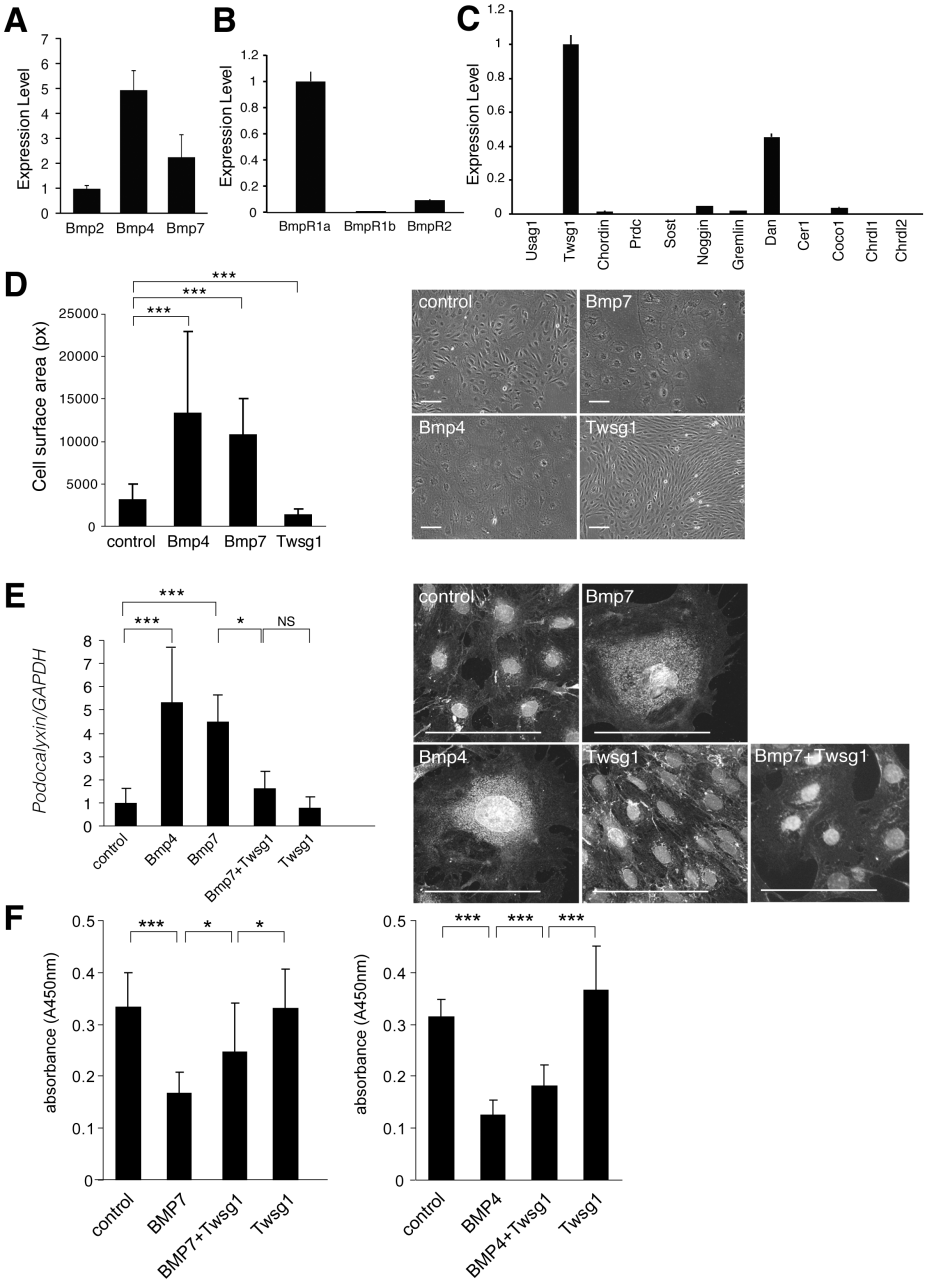
Expression and function of Bmps and Bmp antagonists in cultured podocytes. (A–C) Murine podocytes cultured under non-permissive conditions were subjected to quantitative real-time PCR with various primers for Bmps, Bmp receptors and Bmp antagonists using vector control (see method)(n = 3). (A) Expression of Bmps in cultured podocytes. The expression of each Bmp normalized by vector control was shown relative to that of Bmp2. (B) Expression of Bmp receptors in cultured podocytes. Expression of Bmp receptor type Ib and Bmp receptor type II normalized by vector control was shown relative to that of Bmp receptor type Ia. (C) Expression of Bmp antagonists in cultured podocytes. The expression of Bmp antagonists normalized by vector control was shown relative to that of Twsg1. (D–E) The effects of Bmps and Twsg1 administration on cultured podocytes. Podocytes cultured under non-permissive conditions were stimulated with Bmp and Twsg1, and incubated for an additional 14 days. Recombinant Bmp7 (300 ng/ml), Bmp4 (300 ng/ml), Twsg1 (300 ng/ml) were added to the cultured podocytes at day 1 and day 7. (D) Morphological changes in podocytes after the administration of Bmp4, Bmp7 and Twsg1. Surface area (pixel) of each podocyte was analyzed (n = 30 in each group). (E) The expression of podocalyxin in podocytes treated with Bmp4, Bmp7, and Twsg1. The results of quantitative real-time PCR analysis (n = 7) and representative immunostaining were shown. (F) Proliferation assay of podocyte in the presence of Bmp and Twsg1. We utilized recombinant Bmp7 (300 ng/ml), Bmp4 (300 ng/ml), and Twsg1 (300 ng/ml). (n = 11 in Bmp7 experiment, and n = 8 in Bmp4 experiment) Data are presented as means ± SD. *p<0.05, ***p<0.001; NS, no significant difference. Scale bars: 100 µm.

Cell surface area was analyzed in 30 cells in each group. Podocytes cultured under non-permissive condition for 7 days were subjected for real-time RT-PCR analysis (n = 3 in Figure 1A–C, and n = 7 in Figure 1E).

### Cell proliferation assay

Cell proliferation was measured using BrDU incorporation assay with Cell Proliferation ELISA, BrDU (colorimetric) kit (Roche Molecular Biochemicals) according to the manufacturer's instruction. Briefly, murine podocytes were seeded at a concentration of 2.4×10^2^ cells/well and were incubated under non-permissive conditions. Recombinant proteins were administered after 24 hours, and podocytes were incubated for additional 5 days. We utilized recombinant Bmp7 (300 ng/ml), Bmp4 (300 ng/ml), and Twsg1 (300 ng/ml). After the incubation, BrdU was administered and the cells were re-incubated for additional 24 hours. After removing culture medium, the cells were fixed and analyzed according to the manufacturer's instruction. (n = 11 in Bmp7 experiment, and n = 8 in Bmp4 experiment)

### Quantification of mRNA by real-time RT-PCR

Real-time RT-PCR was performed with a 7700 Sequence Detection System (Applied Biosystems, Foster City, CA, USA) as previously described[24]. To compare the expression levels of different genes in real-time PCR, we set the standard curve with the plasmid encoding each gene at various concentrations and analyzed the copy number of each gene contained in kidney cDNA[24].

The sequences of the primers were as follows:

Chrdl1:TGGTTTACTGTGTGAACTGCATCTG, GGAATATGCACGGGTGAAAGG

Chrdl2:GATATACTGCGTGCGCTGTACCT, TTGCTGTGGCTCCATCACA

Bmp2: TCGACCATGGTGGCCGGGACCCG, TGTTCCCGGAAGATCTGGAGT

Bmp4: CCGAGCCAACACTGTGAGGAGTTTCC, GGATGCTGCTGAGGTTGAAGAGG

Bmp7: GGATGCTGCTGAGGTTGAAGAGG, TCAGGTGCAATGATCCAGTCC

BmpR1a: TTGACCAGTCCCAAAGCTCTG, CACGCCATTTACCCATCCAT

BmpR1b: CCTGATGCGGCATGAGAATA, TTTGCGTCT AAGGTGGTGGAT

BmpR2:CAACACCACTCAGTCCACCTCAT, CATAAGGCGACTATCAAAACAGCTAA

Podocalyxin: CAGTCAAAGCGTCCTTCAAGCC, TTCATGTCACTGACTCCGGCCT

Podocin: AACCACCATGAAGCGCCTCTT, GGTCACTGCATCTAAGGCAACC

Nephrin: TTCGTCTTGTCGTCCGATTTG, CCCCATTTTTGGTCCAAGTGA

Primer sequences for Bmp antagonists were shown in the previous article[24].

### Immunofluorescence of podocytes

Cells were grown on collagen I-coated glass coverslips and fixed with acetone. Nonspecific binding was blocked with 3% bovine serum albumin plus species-control IgG. The primary antibody utilized was anti-podocalyxin antibody, which was generated according to the method described previously[37]. The specificity of the antibody was confirmed by immunoblotting using anti-podocalyxin mAb 5A (kind gift from Prof. Aaro Miettinen, University of Helsinki, Helsinki, Finland) as a positive control.

### Podocyte injury model

Recombinant LMB2 (1.25 ng/g body weight) was intraperitoneally administered to *NEP* mice to induce podocyte injury as previously described[34]. Glomeruli of *NEP* mice were collected at day 0, day 3 and day 7 of LMB2 administration utilizing the magnetic bead method[38], and were subjected to real-time RT-PCR. (n = 3)

Four days after the injection, urine was collected and analyzed for albumin excretion. Seven days after the injection, *NEP* mice were euthanized, and tissues and blood samples were collected for further analysis[34]. Blood samples were analyzed for serum albumin, total cholesterol, creatinine and blood urea nitrogen levels in 30 LMB2-injected *Twsg1^+/+^:NEP25* mice, 24 LMB2-injected *Twsg1^LacZ/LacZ^:NEP25* mice and 13 control mice. Kidney samples of 15 LMB2-injected *Twsg1^+/+^:NEP25* mice, 9 LMB2-injected *Twsg1^LacZ/LacZ^:NEP25* mice and 6 control mice were subjected to real-time PCR analysis of nephrin, podocin and podocalyxin.

### LacZ staining

LacZ staining was performed as described previously[25]. For double staining, immunostaining with anti-laminin antibody (Biomedical) was performed after LacZ staining.

### Assessment of albuminuria

The mice were placed in metabolic cages, and urine was collected over a 24-hour period as described[26]. During the urine collection, mice were allowed free access to food and water. Urinary albumin concentration was measured using the Albuwell M assay kit (Exocell Inc. Philadelphia, PA, USA) for 15 *Twsg1^+/+^:NEP25* mice and 17 *Twsg1^LacZ/LacZ^:NEP25* mice.

### Histological analysis

The kidneys were fixed in Carnoy's solution and embedded in paraffin. Sections (2-µm thick) were stained with PAS for routine histological examination, and the degree of morphological change was determined for 26 *Twsg1^+/+^:NEP25* mice and 23 *Twsg1^LacZ/LacZ^:NEP25* mice by experienced pathologists who were blinded to the genotypes. The numbers of glomeruli counted in each mouse were 90±14 in *Twsg1^+/+^:NEP25* mice, and 97±16 in *Twsg1^LacZ/LacZ^:NEP25* mice, and the numbers were not significantly different between two genotypes.

Glomerular injuries were classified into four categories according to the percentage of hemorrhagic glomeruli, sclerotic glomeruli, and cellular crescents: No lesion, 0%; mild, <10%; moderate, 10%–25%; severe, >25%. Tubulointerstitial injuries were also classified into four categories according to the percentage of injured area: No lesion, 0%; mild, <10%; moderate, 10%–25%; severe>25%.

### Statistical analysis

Data are presented as means ± SD. Statistical significance was assessed by Student's *t* test for two-group comparisons. *P*<0.05 is considered significant. For the histological analysis of podocyte injury model, statistical significance was assessed using the Kruskal-Wallis test.

## Results

### Twsg1 is the most abundant Bmp antagonist in cultured podocytes

Expression of Bmps, Bmp receptors and Bmp antagonists was first examined using murine immortalized podocyte cell lines (kind gifts from Professors P. Mundel and S.J. Shankland)[36]. We demonstrated that Bmp ligands as well as Bmp type I and type II receptors were expressed in these cell lines (**Figure 1A, B**). Among the known Bmp antagonists, Twsg1 was the most abundantly expressed in podocytes (**Figure 1C**).

### Bmps inhibit proliferation and accelerate differentiation of podocytes, and Twsg1 antagonizes these effects

The podocyte cell lines stably express the temperature-regulated tsA58 mutant T antigen, and proliferate under permissive conditions (33°C supplemented with IFN-γ). Under growth restrictive conditions (37°C without IFN-γ), podocytes stop replicating within 14 days, substantially increase in size, and begin to express podocyte markers such as podocalyxin[36].

The administration of Bmp4 and Bmp7 to the cultured podocytes increased cell size, whereas the administration of Twsg1 decreased cell size (**Figure 1D**). Podocalyxin is a podocyte marker expressed in differentiated podocytes, that plays an essential role in the maintenance of podocyte foot processes[39]. The expression of podocalyxin in cultured podocytes was significantly increased by the administration of Bmp4 and Bmp7, whereas the simultaneous administration of Twsg1 antagonized this effect (**Figure 1E**). These data show that Bmp accelerates differentiation of podocytes, whereas Twsg1 attenuates the effects of Bmp. We also demonstrated that the administration of Bmp4 and Bmp7 to the cultured podocytes inhibited cell proliferation, whereas the administration of Twsg1 attenuated the inhibitory effect (**Figure 1F**).

### Bmp signaling pathway in podocyte differentiation

**Figure 2:**
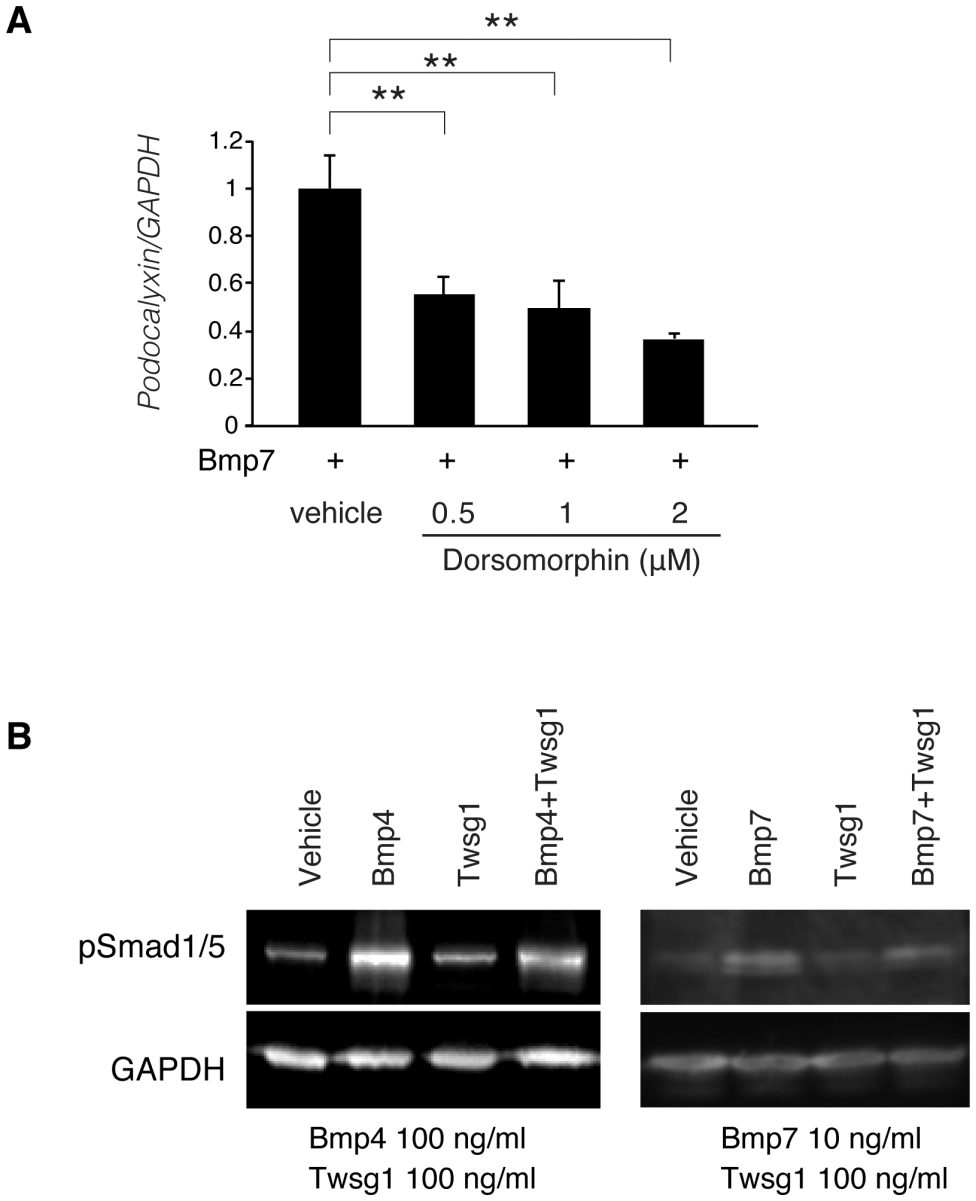
Smad signaling in podocyte differentiation and Twsg1 antagonism. (A) The results of quantitative real-time PCR analysis of podocalyxin in the presence or absence of dorsomprohin. Bmp7 (10 ng/ml) and each concentration of dorsomphin were added to the podocyte. Data are presented as means ± SD. n = 3. **, p<0.01. (B) Western blotting of podocytes treated with Bmp proteins and Twsg1. Podocytes treated with these proteins were subjected for western blotting. Representative data is shown. GAPDH was utilized as a loading control.

The downstream signaling pathways of Bmp include phosphorylation of Smad1/5/8 and other non-canonical intracellular effectors such as JNK and p38. Previously, Mitu *et al.* demonstrated that Smad5 plays an important role in the anti-apoptotic effect of Bmp7 in podocytes cultured under high-glucose conditions[16]. We found that the administration of dorsomorphin[40], [41], a small molecule inhibitor of Bmp-mediated Smad1/5/8 phosphorylation, significantly decreased the expression of podocalyxin (**Figure 2A**), indicating that the stabilization of podocyte differentiation by Bmp is mediated by Smad1/5/8 signaling. We further confirmed that the administration of Twsg1 partially attenuated the phosphorylation of Smad1/5 (**Figure 2B**).

### Twsg1 is expressed in Bowman's capsule, and is additionally expressed in glomerular cells in a podocyte injury model

The expression of Twsg1 in the kidney was analyzed utilizing Twsg1^LacZ/+^ mice, in which β-galactosidase gene was inserted in the locus of Twsg1 gene[33].

**Figure 3:**
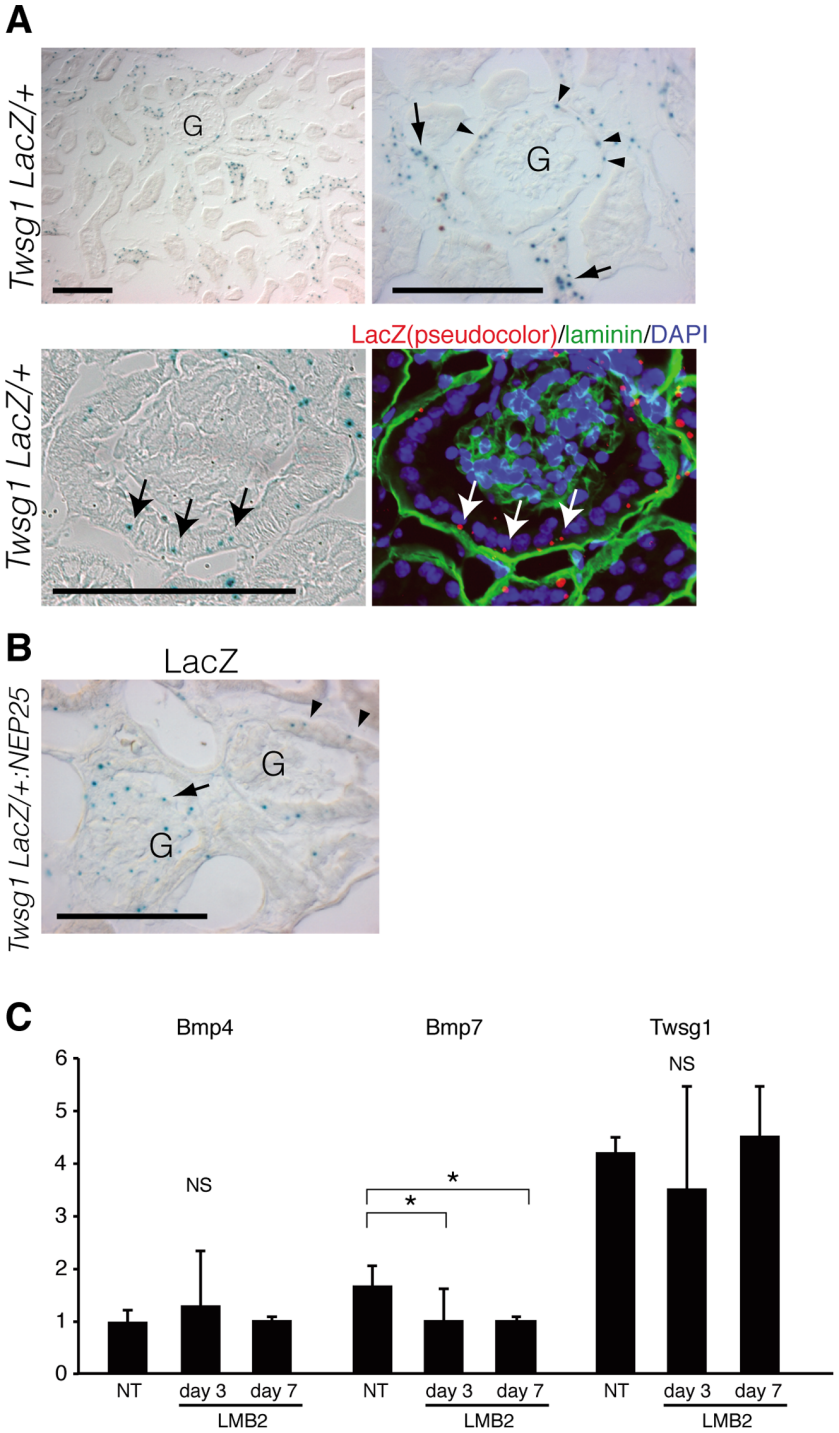
Localization of Twsg1 in healthy and diseased kidney. (A, upper row) X-gal staining of the kidney of *Twsg1^+/LacZ^* mice. Twsg1 was expressed in glomerular parietal cells (PECs)(arrowheads), and distal nephrons (arrows). Twsg1 expression was not observed in podocytes of healthy kidneys. G: glomeruli. (A, lower row) X-gal staining and laminin immunostaining to show the expression of Twsg1 in PECs (arrows). Scale bars: 100 µm. (B) X-gal staining of the kidney of *Twsg1^+/LacZ^:NEP25* mice treated with immunotoxin. LacZ staining emerged within glomerular cells of some glomeruli (arrows), whereas LacZ staining in other glomeruli was confined in PECs (arrowheads). Scale bars: 100 µm. (C) Glomerular RNA of *NEP25* mice was collected before and after the administration of LMB2, and was analyzed for the relative expression of Bmp and Twsg1 by real-time PCR. (n = 3) NT, non treat. Data are presented as means ± SD. * *p*<0.05; NS, no significant difference.

In healthy kidneys, weak LacZ staining was observed in glomerular parietal cells (PECs) and a part of distal nephron, but not in glomerular cells (**Figure 3A**).

We further induced podocyte injury in Twsg1^LacZ/+^ mice, utilizing a transgenic mouse strain expressing human CD25 selectively in podocytes (NEP25 mice)[34]. Injection of an immunotoxin targeting human CD25, anti-Tac(Fv)-PE38 (LMB2)[42], to NEP25 mice induces severe podocyte injury and massive proteinuria.

In Twsg1^LacZ/+^:NEP25 mice injected with LMB2, LacZ staining emerged within glomerular cells in some glomeruli, whereas LacZ staining in other glomeruli was still confined in PECs (**Figure 3B**).

To quantitate the expression of Bmp4, Bmp7 and Twsg1 in glomeruli during this model, we collected glomeruli utilizing magnetic beads method[38], and analyzed the expression of these genes by real-time RT-PCR (**Figure 3C**). The expression of Bmp7 decreased significantly during this disease model, whereas the expression of Bmp4 did not. Although the localization of Twsg1 changed during the disease model, total amount of Twsg1 mRNA was not changed.

### Twsg1 null mice are resistant to podocyte injury

To examine the role of Twsg1 in the progression of podocyte injury, we generated Twsg1 null mice by crossing Twsg1^LacZ/+^ heterozygous mice[33]. Histological analysis as well as renal function was comparble between Twsg1^LacZ/LacZ^ and Twsg1^+/+^ mice (data not shown). We further analyzed the number of podocytes and the expression of podocyte differentiation markers, and did not find significant differences between both genotypes at the baseline (**Figure S1**).

**Figure 4:**
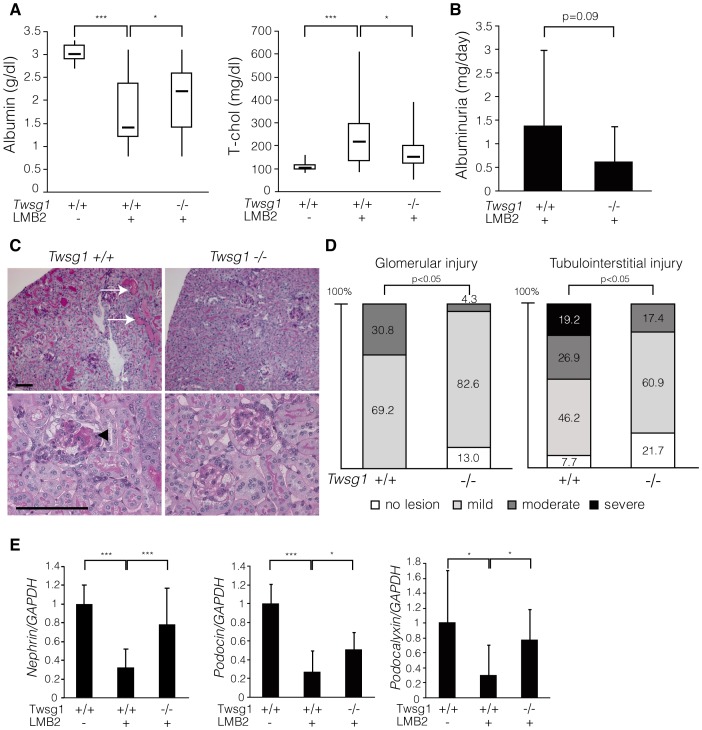
Twsg1 null mice were resistant to podocyte injury. (**A**) Twsg1^+/+^:NEP25 and Twsg1^LacZ/LacZ^:NEP25 mice were injected with immunotoxin and analyzed seven days later. While Twsg1^+/+^:NEP25 mice injected with immunotoxin (Tswg1+/+LMB2+, n = 30) showed decreased levels of serum albumin and increased levels of total cholesterol compared to the controls (Tswg1+/+LMB2-, n = 13), these changes in serum parameters were significantly attenuated in Twsg1^LacZ/LacZ^:NEP25 mice (Tswg1−/−LMB2+, n = 24). Data are given in boxplots, in which the bottom and top of each box represent the 25th and 75th percentile, respectively, and the band near the middle of each box indicates the median. The ends of the whiskers represent the minimum and maximum of all data. (**B**) Urinary albumin levels were analyzed four days after the injection. Twsg1^LacZ/LacZ^:NEP25 mice (Tswg1−/−LMB2+, n = 17) showed lower levels of urinary albumin compared to Twsg1^+/+^:NEP25 mice (Tswg1+/+LMB2+, n = 15), although this difference was not statistically significant. Data are presented as means ± SD. (**C**) Representative histological findings of both genotypes seven days after the injection. Histological changes such as tubular degeneration (arrows) and glomerulosclerosis (arrowhead) were less severe in Twsg1^LacZ/LacZ^:NEP25 mice (Tswg1−/−) than in Twsg1^+/+^:NEP25 mice (Tswg1+/+). Scale bars: 100 µm. (**D**) Glomerular and tubulointerstitial injury was analyzed semi-quantitatively in both genotypes. The severity of both glomerular and tubulointerstitial damage was significantly lower in Twsg1^LacZ/LacZ^:NEP25 mice (Tswg1−/−, n = 23) than in Twsg1^+/+^:NEP25 mice (Tswg1+/+, n = 26). (**E**) Quantitative real-time PCR analysis of podocyte-specific genes in the whole kidneys seven days after the injection. Expression levels of nephrin, podocin and podocalyxin were severely reduced due to the injection of the immunotoxin in Twsg1^+/+^:NEP25 mice (Tswg1+/+LMB2+, n = 15) compared to the controls (Tswg1+/+LMB2-, n = 6), whereas this reduction was significantly attenuated in Twsg1^LacZ/LacZ^:NEP25 mice (Tswg1−/−LMB2+, n = 9). Data are presented as means ± SD. *p<0.05, ***p<0.001.

Next we generated NEP25 transgenic mice deficient in Twsg1 gene (Twsg1^LacZ/LacZ^:NEP25 mice) along with the control littermates (Twsg1^+/+^:NEP25 mice) and administered the abovementioned immunotoxin to both genotypes. While Twsg1^+/+^:NEP25 mice exhibited severe nephrotic syndrome characterized by massive proteinuria, hypoalbuminemia and hyperlipidemia, Twsg1^LacZ/LacZ^:NEP25 mice showed milder hypoalbuminuria and hyperlipidemia (**Figure 4A**). The severity of albuminuria tended to be lesser in Twsg1^LacZ/LacZ^:NEP25 mice than in their control littermates, although the difference was not statistically significant (**Figure 4B**). Renal function was maintained in both genotypes (**Figure S2**).

Histological analysis demonstrated glomerular injury and tubulointerstitial damage in Twsg1^+/+^:NEP25 mice, and quantitative analysis demonstrated milder histological changes in Twsg1^LacZ/LacZ^:NEP25 mice compared to Twsg1^+/+^:NEP25 mice (**Figure 4C, D**), whereas the expression of hCD25 was comparable between groups (Figure **S3**). Furthermore, the expression of podocyte markers in the kidneys of Twsg1^+/+^:NEP25 mice was reduced by the administration of the immunotoxin, whereas this reduction was significantly attenuated in Twsg1^LacZ/LacZ^:NEP25 mice (**Figure 4E** **, Figure S4**).

Taken together, these findings show that Twsg1 null mice were more resistant to podocyte injury than their control littermates were.

## Discussion

Twsg1 is known for its context-dependent anti- and pro-Bmp functions: Twsg1 inhibits Bmp activity, either directly or indirectly, in some contexts[28], [29], [31], [43], while enhancing Bmp signaling in others[27], [33], [44]. Even in the kidney, Twsg1 functions as anti- and pro-Bmp factor: Twsg1 shows pro-Bmp activity enhanced by Crossveinless2 (Cv2) during kidney development[45], whereas Twsg1 inhibits Bmp signaling together with Chordin-like 1 (Chrdl1) in tubular epithelial cells of adult kidney[46].

In this manuscript, we demonstrated that the administration of Bmp7 induces the differentiation and inhibits the proliferation of podocytes, whereas the simultaneous administration of Twsg1 antagonizes these effects of Bmp7. Although the mechanisms determining the direction of Twsg1 function are unclear, the results of this study support the idea that Twsg1 functions as an anti-Bmp factor in podocytes. Although Twsg1 is considered to form a trimer including Bmp and Chordin, Chrdl1, Chrdl2 in some context[27], [46], the trimer formation is less likely in podocytes because the expression of Chordin, Chrdl1, Chrdl2 was very low (**Figure 1C**).

We further showed that the stabilization of podocyte differentiation by Bmp is, at least in part, mediated through pSmad1/5/8 signaling, and that this pathway is partially blocked by Twsg1. However, the inhibitory effect of Twsg1 on the phosphorylation of Smad is moderate compared to the inhibitory effect of Twsg1 on podocyte differentiation. This discrepancy indicates the contribution of other signaling pathways in the inhibitory effect of Twsg1 on podocyte differentiation.

We also confirmed that Twsg1 is expressed in the glomerular parietal epithelial cells in healthy kidneys by the double staining of LacZ and laminin (the marker of basement membrane), and additionally expressed in glomerular cells in a podocyte injury model, although the cell type expressing Twsg1 in injured glomeruli is still unclear due to weak LacZ staining. Several groups recently reported that glomerular parietal epithelial cells (PECs) migrate onto glomerular tufts and differentiate into podocytes after glomerular injury[47], [48]. LacZ positive cells in injured glomeruli of *Twsg1^LacZ/+^:NEP25* mice might be the migrating PECs, which originally express *Twsg1/β-galactosidase*. Another possible explanation is that injured podocytes begin to express *Twsg1/β-galactosidase*. This idea is supported by the fact that cultured podocytes expressing Twsg1. In either way, it is plausible that the expression of Twsg1 in the glomeruli accelerates podocyte injury by inhibiting the renoprotective action of Bmp signaling. Reduction of Bmp7 expression in the glomeruli of *NEP* mice should also contribute to the attenuation of Bmp signaling in the glomeruli (**Figure 3C**).

We further demonstrated that *Twsg1* null mice are resistant to podocyte injury, possibly due to the enhanced Bmp signaling in podocytes. Although Twsg1 is also known to inhibit T cell activation and plasma cell production[49], [50], the explanation that Twsg1 deletion in the immune system interferes with the progression of podocyte injury is less likely to be correct given that inflammatory cell infiltration not prominent in this model (data not shown).

Although several reports have indicated that pharmacological doses of Bmp7 inhibit renal injury in animal models, the systemic administration of Bmp7 causes undesired side effects in other tissues, because Bmp receptors are widely expressed[21]. Previously, we demonstrated that a product of *uterine sensitization-associated gene-1* (USAG-1), a novel Bmp antagonist expressed in the distal tubules of the kidney, plays a critical role in modulating the renoprotective action of Bmp, and that inhibition of USAG-1 is a promising potential novel treatment for various renal diseases[23], [24], [26]. Our current data indicate that Twsg1 attenuates the protective effect of Bmp7 on podocyte maintenance, and that inhibition of Twsg1 might be beneficial in the treatment of podocyte injury. These results led us to postulate the presence of cell type-specific Bmp modulators and the precise regulation of Bmp signaling in the kidney. Future studies improving our understanding of Bmp regulators are expected to provide additional support for a therapeutic approach based on suppression of Bmp regulators rather than the systemic administration of Bmp.

## Supporting Information

Figure S1 (A) Podocyte numbers were analyzed by counting WT1-positive podocytes in both groups. 38 glomeruli were analyzed in wild-type kidneys, whereas 42 glomeruli were analyzed in Twsg1 null kidneys. (B) The expression of nephrin, podocin, and podocalyxin were analyzed in both groups. Data were normalized by GAPDH and were shown in relative to those of wild type littermates. Data are presented as means ± SD. n = 4. NS, no significant difference.
(TIF)

Figure S2 Serum creatinine and BUN in the mice of Figure 3A. (Tswg1+/+LMB2-: n = 13, Tswg1+/+LMB2+: n = 30, Tswg1−/−LMB2+: n = 24) Data are given in boxplots, in which the bottom and top of each box represent the 25th and 75th percentile, respectively, and the band near the middle of each box indicates the median. The ends of the whiskers represent the minimum and maximum of all data. NS, no significant difference.
(TIF)

Figure S3 Real-time PCR of hCD25 in the samples of Figure 3E. Data were normalyzed to those of GAPDH. Data are presented as means ± SD. NS, no significant difference.
(TIF)

Figure S4 (A) Representative immunostaining of podocalyxin in Twsg1 wild-type and null mice. (B) Representative immunostaining of podocalyxin in Twsg1 +/+:NEP mice and Twsg1−/−:NEP mice with or without LMB2 administration. Scale bars: 100 µm.
(TIF)
