# Supplementary figures and images for: Twisted Gastrulation, a BMP Antagonist, Exacerbates Podocyte Injury
Source: PLoS One. 2014 Feb 25;9(2):e89135. doi: 10.1371/journal.pone.0089135 (PMC3934867; doi:10.1371/journal.pone.0089135)

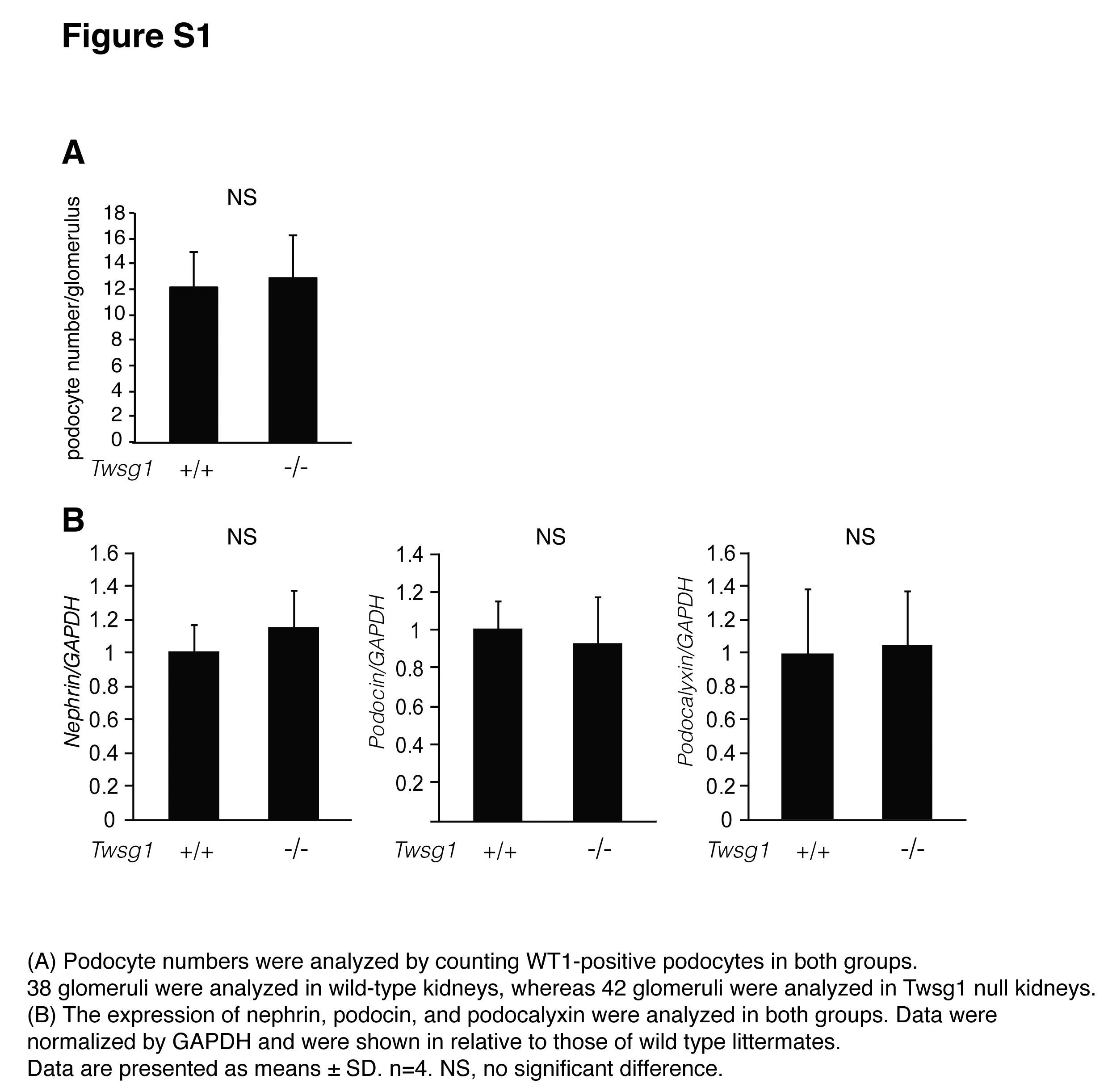

Supplement: Figure S1 — (A) Podocyte numbers were analyzed by counting WT1-positive podocytes in both groups. 38 glomeruli were analyzed in wild-type kidneys, whereas 42 glomeruli were analyzed in Twsg1 null kidneys. (B) The expression of nephrin, podocin, and podocalyxin were analyzed in both groups. Data were normalized by GAPDH and were shown in relative to those of wild type littermates. Data are presented as means ± SD. n = 4. NS, no significant difference. (TIF) [file pone.0089135.s001.tif]

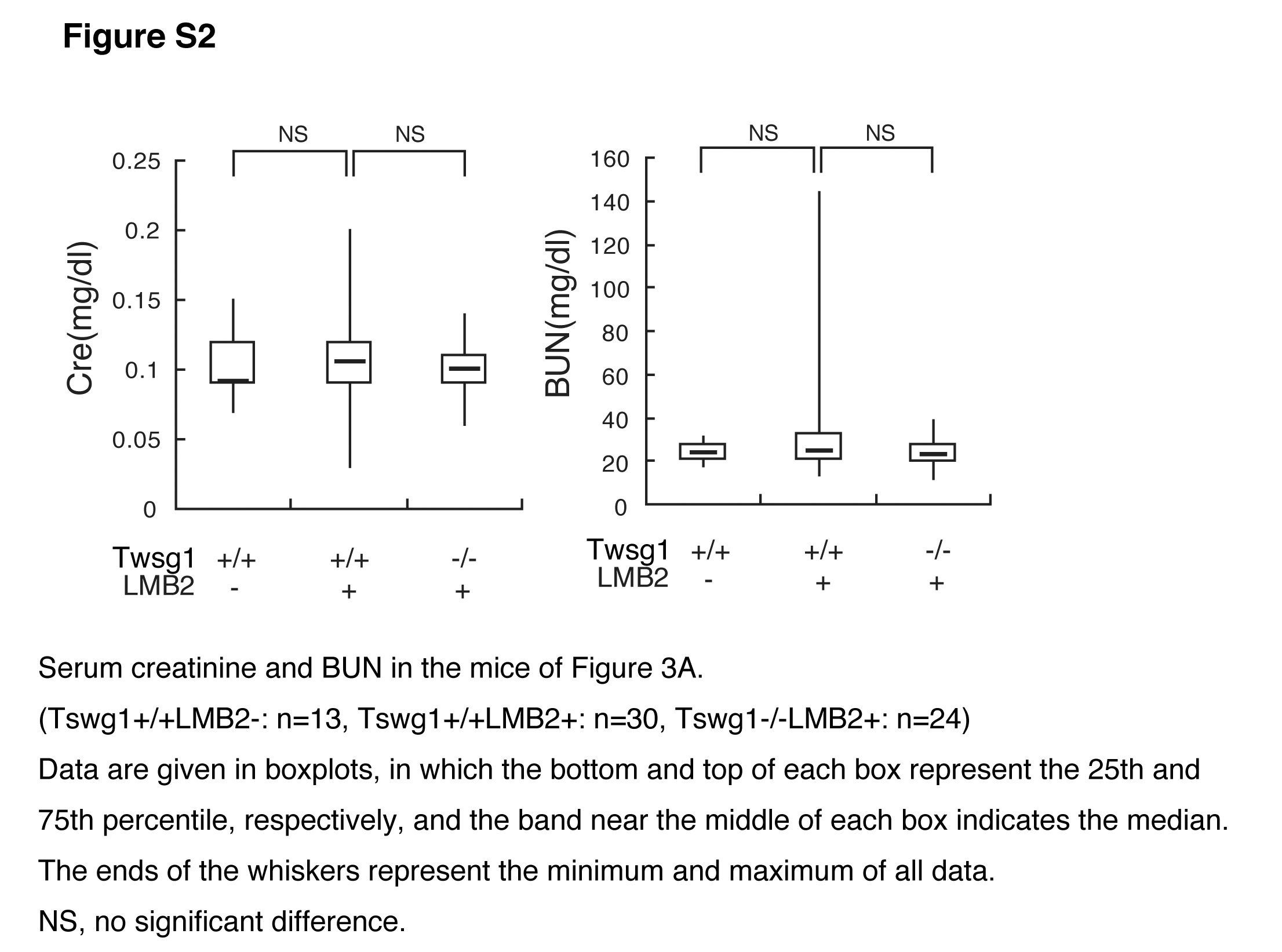

Supplement: Figure S2 — Serum creatinine and BUN in the mice of Figure 3A. (Tswg1+/+LMB2-: n = 13, Tswg1+/+LMB2+: n = 30, Tswg1−/−LMB2+: n = 24) Data are given in boxplots, in which the bottom and top of each box represent the 25th and 75th percentile, respectively, and the band near the middle of each box indicates the median. The ends of the whiskers represent the minimum and maximum of all data. NS, no significant difference. (TIF) [file pone.0089135.s002.tif]

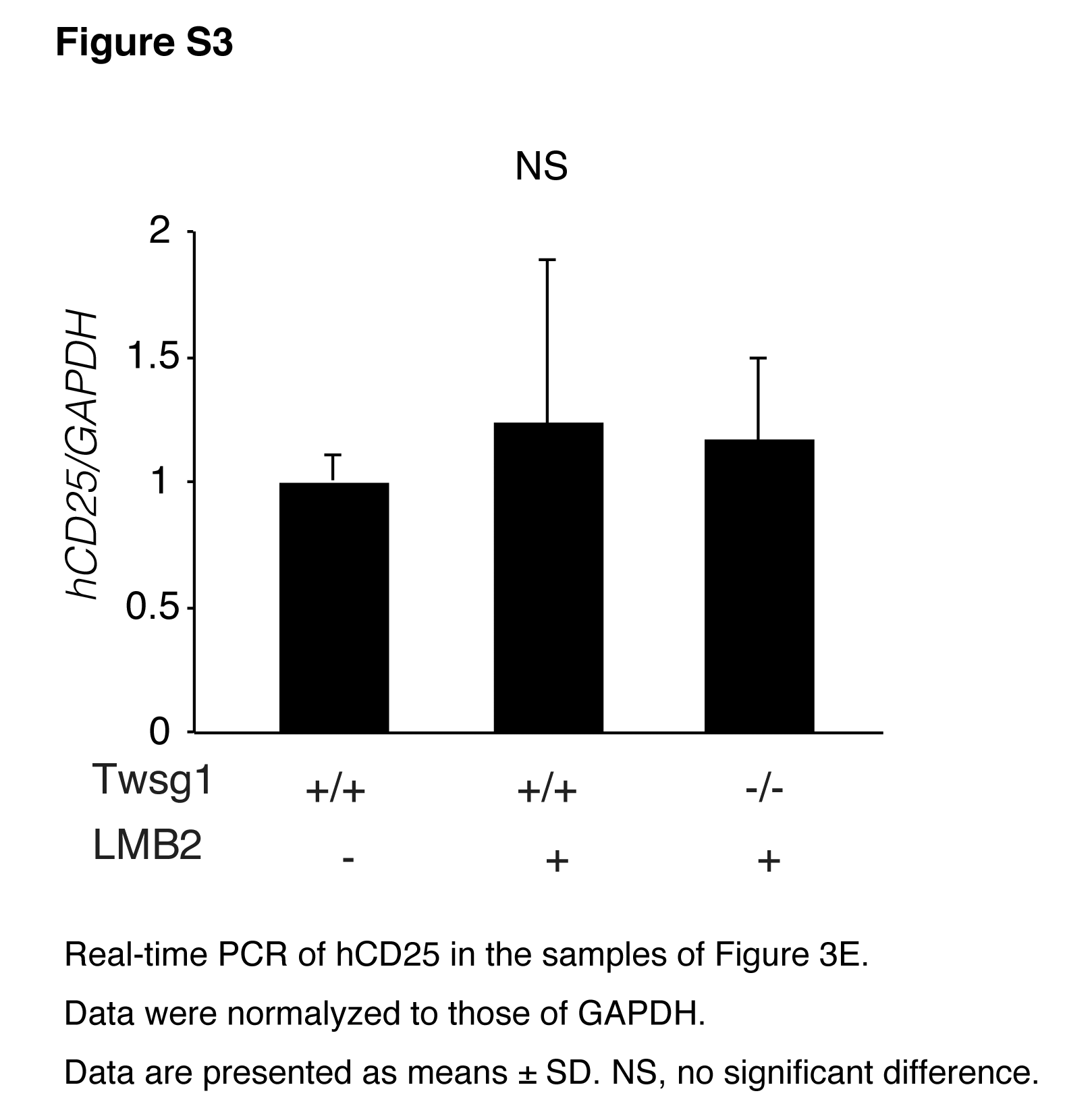

Supplement: Figure S3 — Real-time PCR of hCD25 in the samples of Figure 3E. Data were normalyzed to those of GAPDH. Data are presented as means ± SD. NS, no significant difference. (TIF) [file pone.0089135.s003.tif]

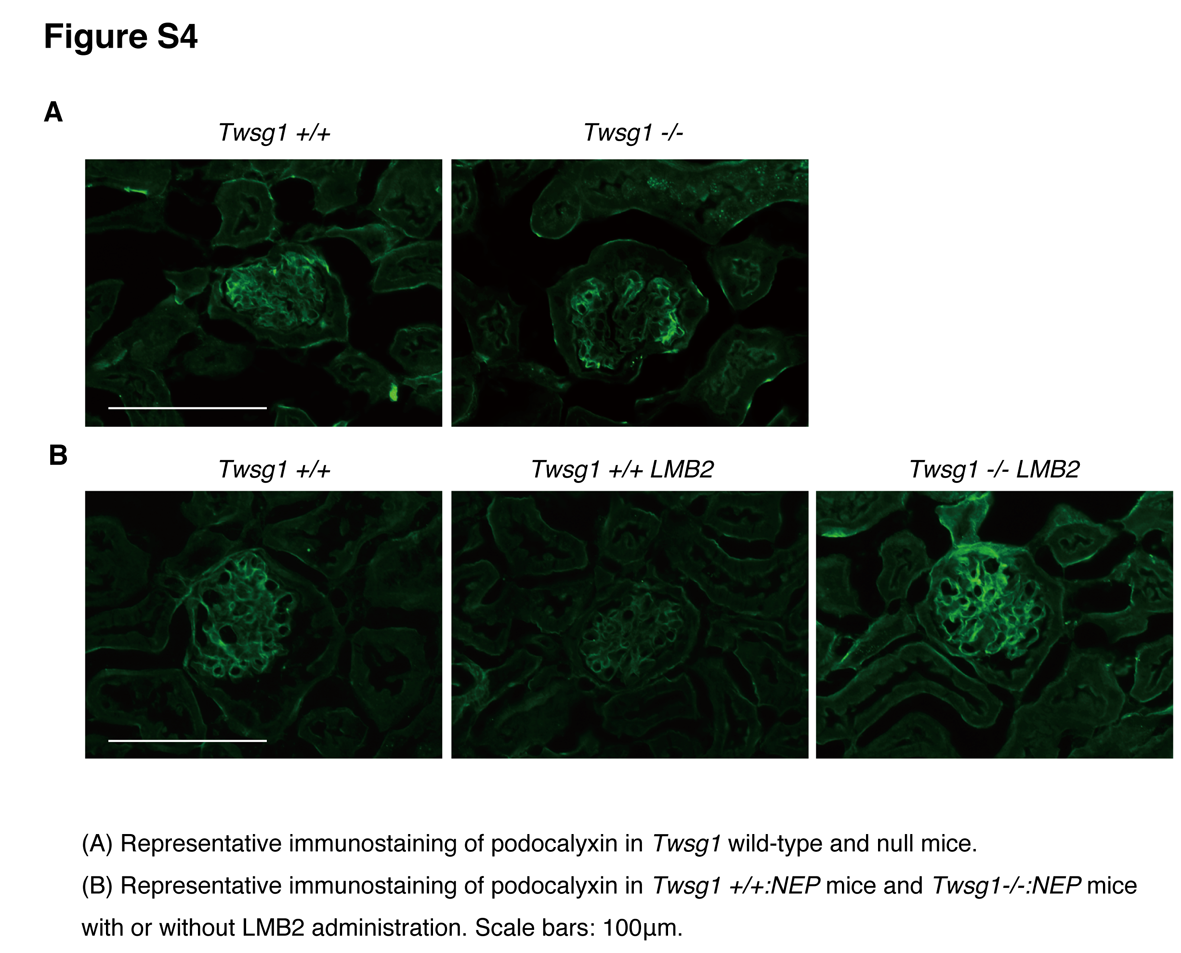

Supplement: Figure S4 — (A) Representative immunostaining of podocalyxin in Twsg1 wild-type and null mice. (B) Representative immunostaining of podocalyxin in Twsg1 +/+:NEP mice and Twsg1−/−:NEP mice with or without LMB2 administration. Scale bars: 100 µm. (TIF) [file pone.0089135.s004.tif]
